# *Hv*RNASET2 Regulate Connective Tissue and Collagen I Remodeling During Wound Healing Process

**DOI:** 10.3389/fphys.2021.632506

**Published:** 2021-02-24

**Authors:** Nicolò Baranzini, Laura Pulze, Gianluca Tettamanti, Francesco Acquati, Annalisa Grimaldi

**Affiliations:** Department of Biotechnology and Life Science, University of Insubria, Varese, Italy

**Keywords:** innate immunity, invertebrates, medicinal leech, ribonucleases T2, collagen, regeneration

## Abstract

Several studies have recently demonstrated that the correct regeneration of damaged tissues and the maintaining of homeostasis after wounds or injuries are tightly connected to different biological events, involving immune response, fibroplasia, and angiogenetic processes, in both vertebrates and invertebrates. In this context, our previous data demonstrated that the *Hirudo verbana* recombinant protein r*Hv*RNASET2 not only plays a pivotal role in innate immune modulation, but is also able to activate resident fibroblasts leading to new collagen production, both *in vivo* and *in vitro*. Indeed, when injected in the leech body wall, which represents a consolidated invertebrate model for studying both immune response and tissue regeneration, *Hv*RNASET2 induces macrophages recruitment, fibroplasia, and synthesis of new collagen. Based on this evidence, we evaluate the role of *Hv*RNASET2 on muscle tissue regeneration and extracellular matrix (ECM) remodeling in r*Hv*RNASET2-injected wounded leeches, compared to PBS-injected wounded leeches used as control. The results presented here not only confirms our previous evidence, reporting that *Hv*RNASET2 leads to an increased collagen production, but also shows that an overexpression of this protein might influence the correct progress of muscle tissue regeneration. Moreover, due to its inhibitory effect on vasculogenesis and angiogenesis, *Hv*RNASET2 apparently interfere with the recruitment of the myoendothelial vessel-associated precursor cells that in turn are responsible for muscle regeneration during wound healing repair.

## Introduction

Correct tissue regeneration, following injuries or wounds, involves different biological processes, such as the scavenge of cellular debris and the activation of several processes such as vasculogenesis, angiogenesis, and stimulation/recruitment of immune cell progenitors. Both in vertebrates and in invertebrates, the accurate coordination of these events is essential to re-establish the original, healthy tissue architecture (Pieper et al., 2002; de Eguileor et al., 2005; Li et al., 2005; Grimaldi et al., 2010). Of note, after injuries, immunocompetent cells not only take part in the elimination of possible harmful invaders, but also preserve the homeostasis of tissues and organs and their functional integrity through the elimination of debris, thus avoiding the persistence of immunogenic residues that may induce toxicity and a prolonged inflammatory status within the tissue environment (Martin, 1997; Saltzman, 1999; Frantz et al., 2005). In addition, the release of cytokines and growth factors, such as IL-6, TNF-alpha, and bFGF, by phagocytic cells promotes both fibroblasts proliferation and endothelial cells activation, which in turn leads to the development of new blood vessels (Tettamanti et al., 2003b; de Eguileor et al., 2005). Interestingly, the inflammation-mediated regeneration of tissues represents an evolutionarily conserved process, in which the interaction between regenerative events and the immune system has been observed in many vertebrate and invertebrate model systems (Grimaldi et al., 2009; Schorn et al., 2015; Godwin et al., 2017; Tasiemski and Salzet, 2017; Malagoli, 2018).

In order to investigate these processes, the medicinal leech *Hirudo verbana*, whose use in experimental biology entails minimal ethical concerns and is not regulated, is proposed here to achieve novel information about the mechanisms related to tissue regenerative processes (Grimaldi et al., 2011; Baranzini et al., 2017, 2020b; Girardello et al., 2017). Due to its relatively simple anatomy and small body size, the effects of a wide range of experimental stimuli are easily detected in this animal model in a very short period of time and throughout the entire muscular body wall. For instance, the whole wound healing process is easily detectable after surgical lesions, avoiding any equivocal evaluations, and the subsequent muscular regeneration involves well-established and evolutionary conserved cellular and molecular effectors (Grimaldi et al., 2010). Of note, as already reported in vertebrates, the processes of vasculogenesis, angiogenesis, fibroplasia, and the activation and migration of immunocompetent/muscle precursors cells represent the main steps implicated in restoring damaged tissues in leeches as well (de Eguileor et al., 1999a, 2001; Tettamanti et al., 2005; Grimaldi et al., 2009).

In leeches, wound healing first triggers an inflammatory phase characterized by a massive migration of both immune cells and fibroblasts toward the lesioned area, in which myofibroblast-like cells promote the formation of a pseudoblastema region (Huguet and Molinas, 1994, 1996; Grimaldi et al., 2011). At the same time, the botryoidal tissue, involved in myelo/erythroid and storage functions and promoting vasculogenesis, angiogenesis, and hematopoietic cells production (de Eguileor et al., 1999b, 2001), undergoes an abrupt transition from a cluster/cord-like structure to a hollow architecture typical of pre-vascular structures, in order to generate new capillary vessels, in whose lumen hematopoietic stem precursor cells (HSPCs) subsequently migrate. Most of these circulating immune precursors are recruited into the wounded area and, after leaving the bloodstream, extravasate into the connective tissue (Grimaldi et al., 2006). These cells are committed toward a myogenic or macrophage differentiation pathway by the expression of specific cytokines present in the extracellular matrix (ECM). As in vertebrates, we have previously identified a plethora of cytokines and growth factors in injured leech tissues, such as VEGF (vascular endothelial growth factor), and FGFb (fibroblast growth factor), which support neovessel formation, immune/fibroblast cells recruitment, and proliferation and tissue remodeling by establishing a crosstalk between the innate immune response and tissue regeneration (Grimaldi et al., 2004; Tettamanti et al., 2004, 2005; Baranzini et al., 2020b). In particular, FGFb induces fibroblast proliferation that in turn triggers new collagen synthesis, thus producing a fundamental scaffold for the immune cell migration, the correct orientation of new vessels, and the regeneration of damaged tissues (de Eguileor et al., 2004; Tettamanti et al., 2004). Indeed, collagen is not just a structural component of the ECM, but plays a crucial role in the modulation of different cell functions, including adhesion, growth, and differentiation (Birk and Zycband, 1994; Tettamanti et al., 2003b, 2005). VEGF signaling in leeches modulates vessels migration, proliferation, and survival of both endothelial and myogenic precursors cells within areas of regenerating tissues (Grimaldi et al., 2009, 2010). The different myogenic and endothelial fates of these precursor cells seem to be due to differential responses to VEGF concentrations. The exposure to a sustained and continuous source of VEGF maintains the proliferation and undifferentiated phenotype of hematopoietic CD34^+^ precursor cells. The proliferation stimulus diminishes when the VEGF concentration decreases in the ECM surrounding the muscle fibers, permitting these hematopoietic/endothelial precursor cells to differentiate into muscle fibers.

Given the importance of angiogenesis, fibroplasia, and the innate immune response in the organization and homeostasis of tissues, further research is necessary to elucidate how these processes are interconnected and regulated by both cellular and molecular mechanisms. In this context, recent studies demonstrated that, in *H. verbana*, the T2 ribonuclease *Hv*RNASET2, besides modulating the inflammatory process by recruitment of immune cells in the injured/infected area, is also involved in ECM remodeling and stimulates the production of new collagen both *in vivo* and *in vitro* (Baranzini et al., 2019, 2020b). When injected in the leech body wall, *Hv*RNASET2 induces fibroplasia, connective tissue remodeling, and ECM reorganization, also promoting macrophages recruitment to the stimulated area (Baranzini et al., 2019). Interestingly, these results are supported by *in vitro* experiments conducted on human MRC5 fibroblast cells, in which *Hv*RNASET2 induces the synthesis of new collagen I (Baranzini et al., 2020b).

To better define the direct engagement of *Hv*RNASET2 in tissues reorganization and to obtain novel information about its biological role in a more physiological context, wounded leeches were treated with the recombinant r*Hv*RNASET2 enzyme at different timings, evaluating the ability to stimulate collagen I expression, ECM remodeling, and regeneration of injured tissues.

## Materials and Methods

Leeches (*H. verbana*, Annelida, and Hirudinea, from Ricarimpex, Eysines, France) measuring 10 cm were kept in lightly salted water (NaCl 1.5 g/l) in aerated tanks and kept in an incubator at 20°C. Uninjured and treated animals were anaesthetized by immersion in a 10% ethanol solution until they appeared completely asleep, before being dissected to remove the body wall region of interest.

Leeches were randomly divided into four separate experimental groups (three individuals for each time point) and submitted to various protocols and treatments.

Group 1 included uninjured leeches.

Group 2 included control leeches surgically injured with a razor, immediately injected once, in the same area, with 100 μl of sterilized PBS consisting of 138 mM of NaCl, 2.7 mM of KCl, 4.3 mM of Na2HPO4, and 1.5 mM of KH2PO4, (pH 7.4) to demonstrate that the injection of a vehicle solution alone did not exert a significant effect on wound healing. After 24, 48, 72 h, and 1 week, tissues were collected and fixed as described below. For each timing, experiments were performed in triplicate.

Group 3 included treated leeches surgically injured with a razor and immediately injected with 100 μl of sterilized PBS containing 100 ng of recombinant protein r*Hv*RNASET2, cloned and purified in the yeast *Pichia pastoris* as already described in other studies (Baranzini et al.,2020a,b), in order to verify the effects of the *H. verbana* T2 recombinant protein during the different phases of wound healing. After 24, 48, 72 h, and 1 week, tissues were collected and fixed. For each timing, experiments were performed in triplicate.

### Embedding Tissue in Epoxy Resin for Morphological Analysis at Light and Transmission Electron Microscopy

Specimens of approximately 0.4 mm in thickness and 0.6 mm in length were dissected from the treated leech body wall area and immediately fixed for 2 h in 4% glutaraldehyde in 0.1 M cacodylate buffer at pH 7.4 (de Eguileor et al., 2001; Tettamanti et al., 2003a). After several washes in the same buffer, samples were postfixed for 1 h with 1% osmium tetroxide in cacodylate buffer 0.1 M at pH 7.4, dehydrated in a standard serial ethanol scale (70%, 90%, and absolute), and embedded in an Epon Araldite 812 mixture (Sigma-Aldrich, Milan, Italy). Sections for light microscopy (0.70 μm in thickness) were obtained with a Reichert Ultracut S ultratome (Leica, Wien, Austria), colored by the conventional methods with crystal violet and basic fuchsin [according to Moore et al. (1960)], and subsequently observed under the light microscope Nikon Eclipse (Ni, Nikon, Tokyo, Japan). Data were recorded with a DS-5 M-L1 digital camera system (Nikon). From the same samples, we obtained ultrathin sections (80 nm in thickness) with a Reichert Ultracut S ultratome (Leica) that were placed on copper grids (300 mesh, Sigma-Aldrich, Milan, Italy), counterstained by uranyl acetate and lead citrate, and observed with a Jeol 1010 EX transmission electron microscope (TEM) (Jeol, Tokyo, Japan). Data were recorded with a MORADA digital camera system (Olympus, Tokyo, Japan).

### Embedding Tissue in Paraffin and Masson’s Trichrome Staining

Tissues were fixed in 4% paraformaldehyde for 2 h and then washed three times in PBS solution. Subsequently, samples were dehydrated in an increasing scale of ethanol (30%, 50%, 70%, 90%, 96%, and absolute) and paraffin embedded. Sections (7 μm thick) obtained with a rotary microtome (Jung multicut 2045, Leica) were processed for Trichrome Masson staining (Trichromica kit, Bio Optica, Milan, Italy), as suggested by the datasheet. This coloring technique allows us to observe in blue the collagen and the reticular fibers, while in the red, cell cytoplasm. Images were recorded with a Nikon Digital Sight DS-SM optical Microscope (Nikon, Tokyo, Japan).

### Immunofluorescence Assays

The same samples embedded in paraffin were analyzed by immunofluorescence assays. After paraffin removal, sections were rehydrated with a decreasing scale of ethanol (absolute, 96%, 90%, 70%, and 30%) and then pre-incubated for 30 min in BSA blocking solution (2% Bovine Serum Albumin and 0.1% Tween20 in PBS), which was also used to dilute both the primary and secondary antibodies. Samples were incubated with anti-COL1α1 (rabbit polyclonal, EMD Millipore, raised against peptide mapping at the C-terminal region of human collagen type I) and anti-RNASET2 (rabbit polyclonal, kindly donated by Professor Acquati) primary antibodies, diluted 1:200, for 1 h and after several washes in PBS buffer, they were incubated for 45 min with goat anti-rabbit FITC-conjugated (goat, Jackson ImmunoResearch Laboratories, Baltimore Pike, West Grove–PA, United States) and goat anti-rabbit Cy3-conjugated (goat, Jackson ImmunoResearch Laboratories, Baltimore Pike, West Grove, PA, United States), diluted 1:200, secondary antibodies. Nuclei were counterstained with 4,6-diamidino-2-Phenylinedole (DAPI, 0.1 mg/ml in PBS) for 3 min and slides were mounted with Cityfluor (Cityfluor Ltd., United Kingdom). Negative control experiments were performed omitting primary antibodies.

Double-labeling experiments were performed as previously described (Baranzini et al., 2017), in order to detect the co-localization between bFGFR, RNASET2, and CD34 with COL1α1. The primary antibodies anti-bFGFR (rabbit polyclonal, Santa Cruz Biotechnology, raised against peptide mapping at the C-terminal region of human Flg receptor), diluted 1:100, anti-RNASET2 (rabbit polyclonal), and anti-CD34 (rabbit polyclonal, abcam, raised against peptide mapping from the amino acid 350 to the C-terminal region of human CD34), diluted 1:200, were applied first. After several washes, sections were incubated with the goat anti-rabbit (Cy5)-conjugated (goat, Abcam England) secondary antibody, diluted 1:200. According to Würden and Homberg (1993), in order to block the binding of the second immunofluorescence cycle to the goat anti-rabbit IgGs previously utilized, sections were incubated with rabbit IgG (Jackson ImmunoResearch Laboratories, West Grove, PA, United States) at 1:50 for 2 h. After washing, every slide was treated with the anti-COL1α1 (rabbit, polyclonal, EMD Millipore) primary antibody, diluted 1:200. Procollagen1-α1 primary antibody was subsequently detected with a secondary (FITC)-conjugated goat anti-rabbit antibody (Abcam England), diluted 1:300. Samples were examined with the Nikon Eclipse Ni (Nikon, Tokyo, Japan) light and fluorescence microscope equipped with three different emission filters (360/420 nm for DAPI nuclear staining, 488/525 nm for FITC signals, and 550/580 nm for CY3 signals). Images were recorded with a Nikon digital sight DS-SM (Nikon, Tokyo, Japan) and mounted with Adobe Photoshop (Adobe Systems, San Jose, CA, United States).

### RNA Extraction and qRT-PCR

Tissues extracted from uninjured or wounded and PBS- or *Hv*RNASET2-injected leeches were immediately frozen in liquid nitrogen and then homogenized with a mortar. The obtained homogenates were suspended in 1 mL of TRIzol reagent (Life Technologies) and incubated for 5 min at room temperature. Subsequently, 200 μl of chloroform was added and samples were centrifuged for 15 min at 13,000 rpm at 4°C. Once the different phases separated, 500 μl of the supernatant was recovered and gently mixed with 500 μl of isopropanol. After 10 min of centrifugation at 13,400 rpm, 1 mL of EtOH 75% in DEPC water was added to the precipitated RNA pellet, which was then centrifuged for 5 min at 10,000 rpm, resuspended in 40 μl of DEPC water, and incubated for 10 min at 55°C. The DNA contamination was eliminated by a TURBO DNA-free kit (Thermo Fisher Scientific) and samples were quantified, and purity was evaluated on 1% agarose gel.

In total, 2 μg of RNA were retro-transcribed into cDNA using M-MLV reverse transcriptase (Life Technologies) and qPCR was conducted in triplicate with the iTaq Universal SYBR Green Supermix (Bio-Rad, Hercules, CA) using a 96-well CFX Connect Real Time PCR Detection System (Bio-Rad). qPCR reaction mixtures had a final volume of 15 μl, with 2 μl of diluted cDNA and 10 μM of primers. The primers used for qPCR amplifications were as follows: COL1α1: Fw: 5′-AAGGGAGAGCAAGGAAGACA-3′, Rev: 5′-CCTG GTAAGCCATCAACACC-3′; *Hv*RNASET2: Fw: 5′-GGTCCCAA CTTCTGCACAAAGGAT-3′, Rev: 5′-GTTTGTCCCATTCATG CTTCCAGAA-3′; GAPDH: Fw: 5′-GAAGACTGTGGATGGA CCCT-3′, Rev: 5′-GTTGAGGACTGGGATGACCT-3′. To calculate the relative gene expression, the 2^–ΔΔ*Ct*^ method was performed, with GAPDH as the housekeeping gene. After the initial denaturation, qRT-PCR was performed at 95°C (10 s), 60°C (5 s), and 72°C (10 s) for 39 cycles. Graphs show the COL1α1 and *Hv*RNASET2 quantification (fold change) relative to the GAPDH gene expression.

### Western Blot

Tissues obtained from wounded and PBS- or *Hv*RNASET2-injected leeches were immediately frozen in liquid nitrogen and then homogenized with a T10 basic ULTRA-TURRAX (IKA, Staufen, Germany) in 10 μl of RIPA buffer (50 mM of NaCl, 1% NP-40, 0.5% sodium deoxycholate, 0.1% SDS, 50 mM of Tris–HCl, pH 7.5, protease/phosphatase inhibitors cocktail) per mg of tissue. The lysates were clarified by centrifugation (13,000 rpm at 4°C for 20 min) and protein concentration was determined with the Biuret method. A total of 130 μg of protein extracts were subjected to 8% SDS-PAGE; separated proteins were transferred onto 0.45-μm pore size nitrocellulose membranes (Amersham Protran Premium, GE Healthcare, Chicago, IL, United States). The filters were blocked overnight at 4°C with 5% (w/v) non-fat dried milk in TBS (Tris-buffered saline) and then incubated for 2 h at room temperature with anti-COL1α1 antibody (rabbit, polyclonal) diluted 1:400 in TBS/5% milk; detection of glyceraldehydes 3-phosphate dehydrogenase (GAPDH) was used as loading control. After three washes of 10 min in TBST (Tris-buffered saline containing 0.1% Tween-20), the membranes were incubated for 1 h with horseradish-peroxidase conjugated anti-rabbit (dilution 1:7,500 in TBS/5% milk; Jackson ImmunoResearch Laboratories, West Grove, PA, United States) secondary antibody. The membranes were finally exposed to the enhanced chemiluminescence substrate (LiteAblot PLUS, EuroClone), followed by autoradiography on X-ray film (KODAK Medical X-Ray film, Z&Z Medical, IA, United States). Densitometric analysis was performed with the ImageJ software package^1^. The values are reported as the relative optical density of the bands, normalized to GAPDH.

### Statistical Analyses

Western blot and qPCR experiments were performed in triplicate and data represent the mean values ± SEM. Statistical analyses were performed using GraphPad Prism 7 (GraphPad Software, La Jolla, CA, United States). Statistical differences were calculated by one-way ANOVA followed by Fisher’s *post hoc* test, and *p* < 0.05 was considered statistically significant. In the qPCR assay, means with different asterisks represent a significant difference between wounded and PBS- or r*Hv*RNASET2-injected and untreated leeches at different times. In western blot analyses, means with different letters indicate significant difference between treatments at different times.

## Results

### Morphological Analyses of the Body Wall in Injured and PBS/r*Hv*RNASET2- Injected Leeches

Morphological analyses were carried out on both undamaged and wounded PBS- or r*Hv*RNASET2-injected leeches by means of both light and TEM. Tissues were examined after 24, 48, 72 h, and 1 week from injuries, in order to evaluate the effect of *Hv*RNASET2 on wound healing and tissue regeneration.

**FIGURE 1:**
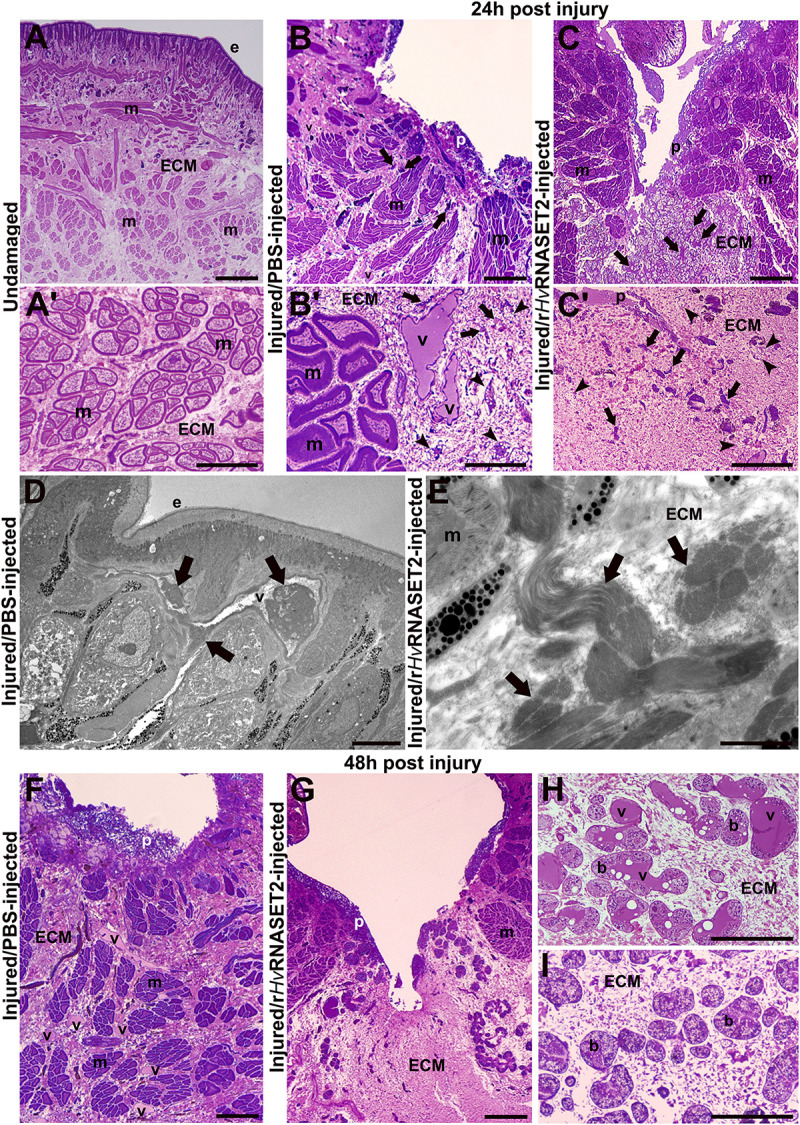
Morphological analyses of the body wall in undamaged (group 1), control (group 2) injured, and PBS–or treated (group 3) r*Hv*RNASET2-injected leeches fixed at 24 and 48 h post-treatment. In undamaged leeches **(A,A’)**, tissue appears predominantly avascular and few resident cells are visible underneath the epithelium (e) and surrounding muscle fibers (m). In control leeches, fixed after 24 h **(B,B’)**, an inflammatory phase arises, and neo-vessels (v), macrophages [arrows in panels **(B,B’)**], and fibroblasts [arrowheads in panel **(B)**] are detectable in the lesioned area. In treated animals **(C,C’)**, numerous immune cells (arrows) and fibroblasts (arrowheads) are visibly immersed in the abundant collagen (ECM). Details of TEM of control **(D)** and treated **(E)** leeches fixed after 24 h. HSPC (arrows) in the lumen of a vessel **(D)** and bundles of collagen fibrils (arrows) are visible **(E)**. In control tissues fixed after 48 h **(F,H)**, numerous blood vessels (v) are evident underneath the pseudoblastema (p) and among muscle fibers (m). Botryoidal tissue (b) appears activated in control animals and pre-vascular structures (v) are visible **(H)**. In treated animals fixed after 48 h **(G, I)**, a huge deposition of ECM in which no vessels are present is evident **(G)**. The botryoidal tissue (b) is in the inactivated form and appears arranged in cords or clusters **(I)**. Bars in panels **(A–C,F,G)** 100 μm; bars in panels **(A’–C’,H,I)** 50 μm; bars in panels **(D,E)** 5 μm.

The organization of the undamaged body wall (Figures 1A,A’) exhibited the typical structure of healthy leeches, in which, underneath the epithelium, few resident cells were present and muscle fibers were immersed in scant connective tissue (Figure 1A’). On the other side, in injured and PBS-injected leeches fixed after 24 h, wound healing was initiated with an inflammatory phase characterized by the migration of numerous macrophages, fibroblasts, and new vessels toward the lesioned area (Figures 1B,B’). After 24 h from injury and r*Hv*RNASET2 injection, a marked ECM deposition and a reduced number of vessels was visible in the lesioned area (Figures 1C,C’). Ultrastructural TEM analyses highlighted the presence of HSPCs in the neo-vessels only in injured PBS-injected leeches (Figure 1D), compared with lesioned and r*Hv*RNASET2-injected animals, in which a large collagen deposition occurred (Figure 1E).

In wounded and control PBS-injected leeches, fixed after 48 h (Figure 1F), the number of neo-vessels increased underneath the pseudoblastema, compared to treated r*Hv*RNASET2-injected animals (Figure 1G). This evidence was supported by the analysis of the botryoidal tissue that in control samples changed its shape from a solid cord to a tubular and pre-vascular structure (Figure 1H), while in treated animals, it appeared inactivated and was arranged in cords or clusters (Figure 1I).

**FIGURE 2:**
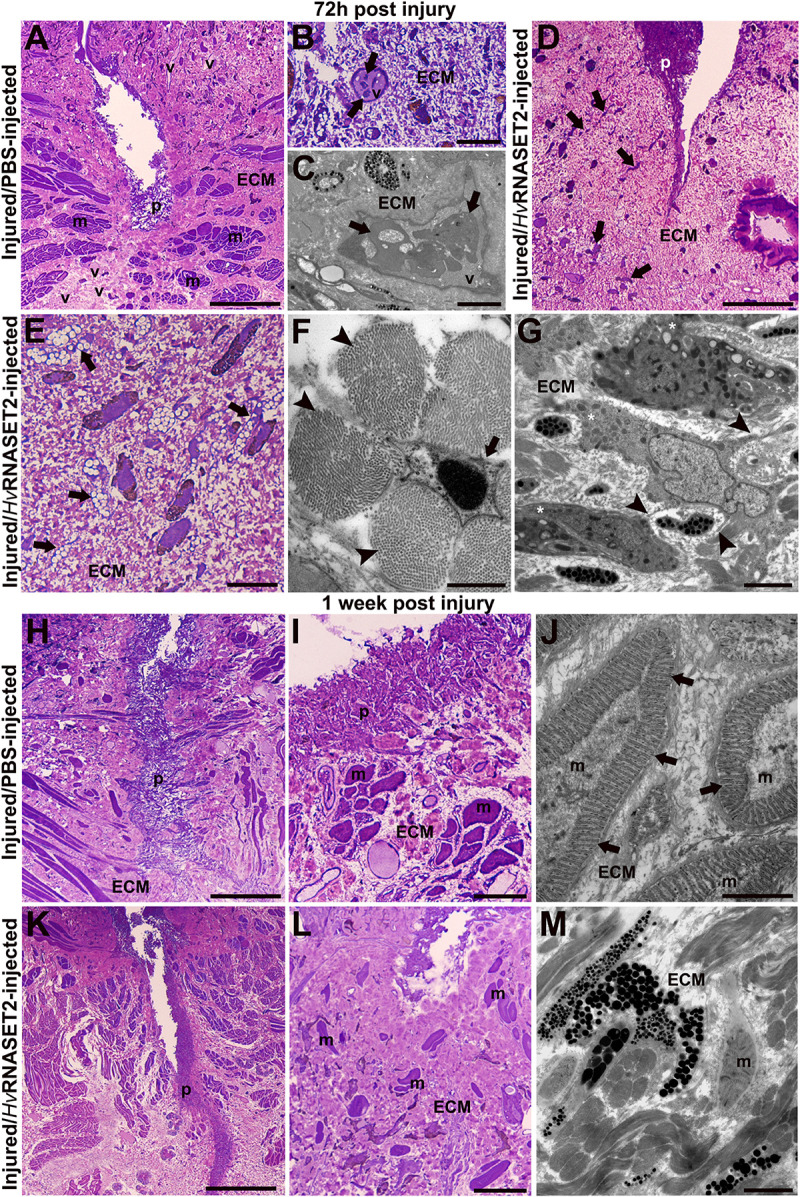
Morphological analyses of the body wall in control (group 2) injured and PBS- or treated (group 3) r*Hv*RNASET2-injected leeches fixed after 72 h and 1 week post-treatment. After 72 h **(A–C)**, in control leeches, numerous vessels (v) and an organized muscle layer are visible **(A)**. Details of light **(B)** and TEM **(C)** microscopes of HSPCs (arrows) in the vessel lumen. In treated leeches **(D–G),** numerous cells (arrows) are evident in the abundant collagen (ECM) underneath the pseudoblastema (p). Activated elongated **(E)** and star-shaped **(F)** fibroblasts (arrows) are involved in new collagen production [arrowhead in panel **(F)**]. Macrophages **(G)** characterized by pseudopodia (asterisks) and membrane blebs (arrowheads). After 1 week, in control animals, the wound is healed and underneath the thick pseudoblastema [p in panels **(H,I)**], a restored muscle layer (m) is evident **(I)**. TEM image showing muscle fibers, with organized sarcomeres (arrows), surrounded by newly synthesized extracellular matrix (ECM) **(J)**. In treated leeches fixed after 1 week, underneath the thick pseudoblastema [p in panels **(K,L)**], a few muscle fibers [m in panels **(L,M)**] are dispersed in the ECM. Bars in panels **(A,D,H,K)** 100 μm; bars in panels **(B,E,I,L)** 10 μm; bars in panels **(G,J,M)** 2 μm; bar in panel **(F)** 500 nm.

In control PBS-injected samples, fixed after 72 h (Figure 2A), a plug was visible under the epithelium. An organized muscle layer was present in the newly synthesized ECM, and HSPCs were clearly detectable in the lumen of neo-vessels (Figures 2B,C). By contrast, in leeches treated with the recombinant *Hv*RNASET2 enzyme a huge deposition of collagen was evident (Figure 2D). Numerous activated fibroblasts appeared elongated (Figure 2E) and star-shaped, characterized by the presence of multiple projections of cytoplasmic laminae stretching toward the extracellular space in which new collagen fibril deposition was identified (Figure 2F).

In addition, macrophages characterized by pseudopodia and blebs (Ma and Baumgartner, 2013; Eom, 2020) were observed in the healing region (Figure 2G).

One week after injury, the complete closure of the wound in control leeches was achieved, due to the formation of a thick pseudoblastema (Figures 2H,I). Furthermore, muscle tissue appeared to be largely repaired, as demonstrated by ultrastructural analysis using TEM showing differentiated muscle fibers surrounded by an abundant collagen matrix and in which contractile material was organized in sarcomeres (Figure 2J). In treated animals, although a thick pseudoblastema was visible, the lesioned area was filled by a massive amount of ECM, while muscle fibers were less represented (Figures 2K–M).

### Masson’s Trichrome Staining

**FIGURE 3:**
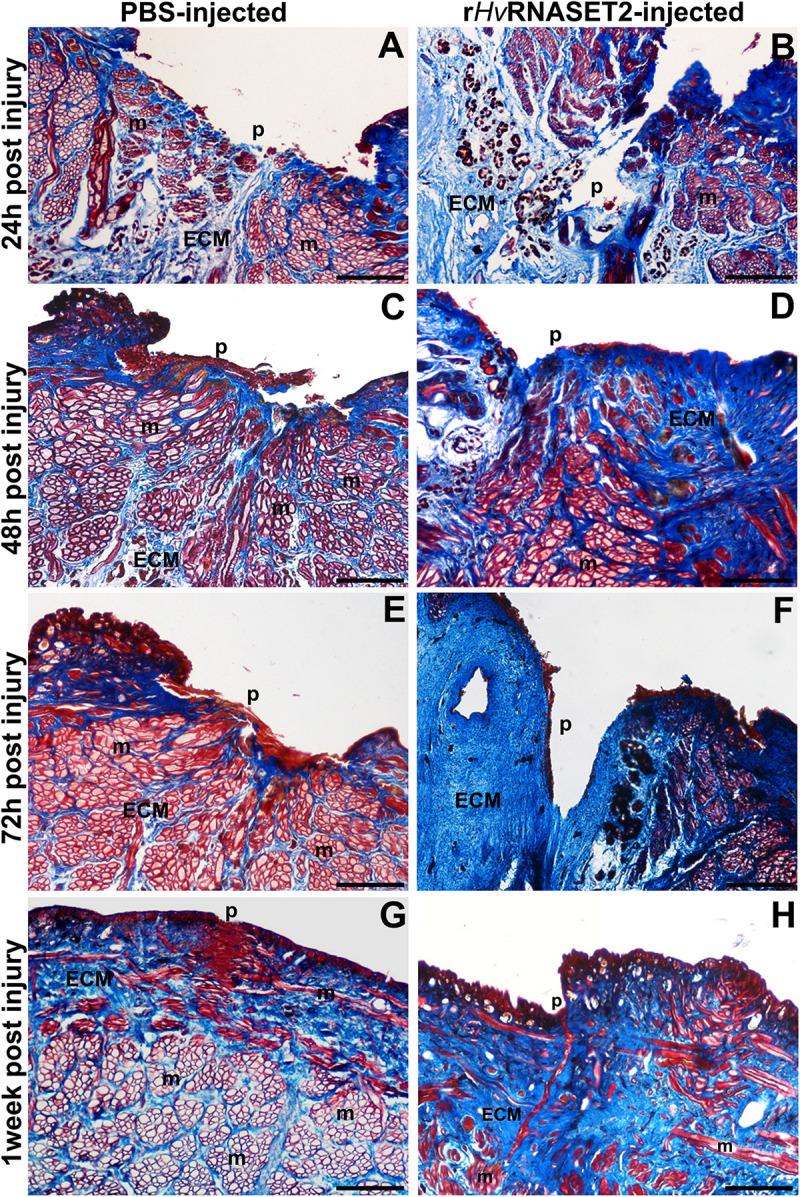
Images of cross-sectioned leech body wall using a light microscope with Masson’s trichrome collagen-specific staining. Collagen is colored in blue (ECM). In leeches treated with PBS **(A,C,E,G)**, collagen fibrils are visible among muscles (m) and underneath the pseudoblastema (p) and lesioned area, while the muscles fiber (m) reorganization is completely regenerated. In r*Hv*RNASET2-injected leeches, an increasing production of collagen is detectable, even after only 24 h **(B)**, which is also maintained after 48–72 h **(D,F)** and 1 week **(H)** from the lesion. Moreover, only a few muscle fibers (m) are recognizable in the newly synthesized compact collagenous scaffold **(G,H)**. p: pseudoblastema, ECM: extracellular matrix. Bars in panels **(A–H)** 100 μm.

Collagen-specific staining (Figure 3) was used to further investigate the effects of *Hv*RNASET2 on ECM remodeling in the healing region. In injured and PBS-injected leeches, the amount of ECM in the injured area and surrounding muscle fibers (Figures 3A,C,E,G) was less than that observed after treatment with r*Hv*RNASET2 (Figures 3B,D,F,H). Indeed, after r*Hv*RNASET2 injection, the amount of newly synthesized ECM increased 48 h after injury and treatment (Figures 3D,F,H). Furthermore, Masson’s trichrome staining clearly showed that the muscle tissue underlying the scar region area was completely regenerated in control animals, whereas in injured r*Hv*RNASET2-treated leeches, only a few muscle fibers were recognizable in the newly synthesized compact collagenous scaffold (Figures 3G,H).

### Immunofluorescence Analyses

**FIGURE 4:**
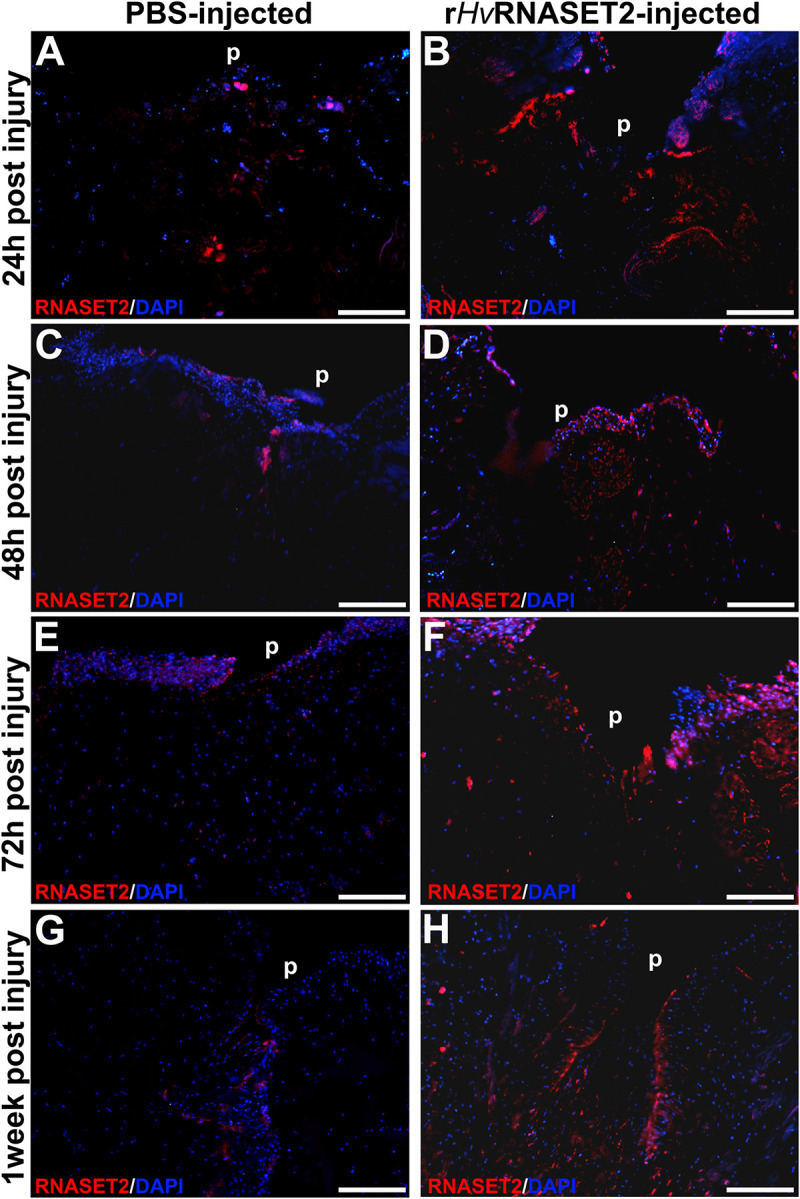
Immunofluorescent analyses of injured and PBS or r*Hv*RNASET2-injected leeches. In PBS-treated animals, an increasing signal for the anti-RNASET2 antibody is detected underneath the pseudoblastema (p) during different time points **(A,C,E,G)**. Instead, in r*Hv*RNASET2-injected leeches, numerous *Hv*RNASET2^+^ cells are already visible after 24 h and the *Hv*RNASET2 signal is higher compared to that observed following PBS treatment in all timings **(B,D,F,H)**. Cell nuclei are stained in blue with DAPI. Bars in panels **(A–H)** 100 μm.

The immunofluorescent assay performed on PBS (Figures 4A,C,E,G) and *Hv*RNASET2-injected (Figures 4B,D,F,H) leeches, showed an increase of *Hv*RNASET2 in the treated groups, as expected. Moreover, the identification of RNASET2^+^ cells, which was particularly evident in the wound healing area and in the underlying connective tissue, suggested that further expression of the endogenous enzyme was triggered on the resident cells by the injected *Hv*RNASET2 at the injured site.

**FIGURE 5:**
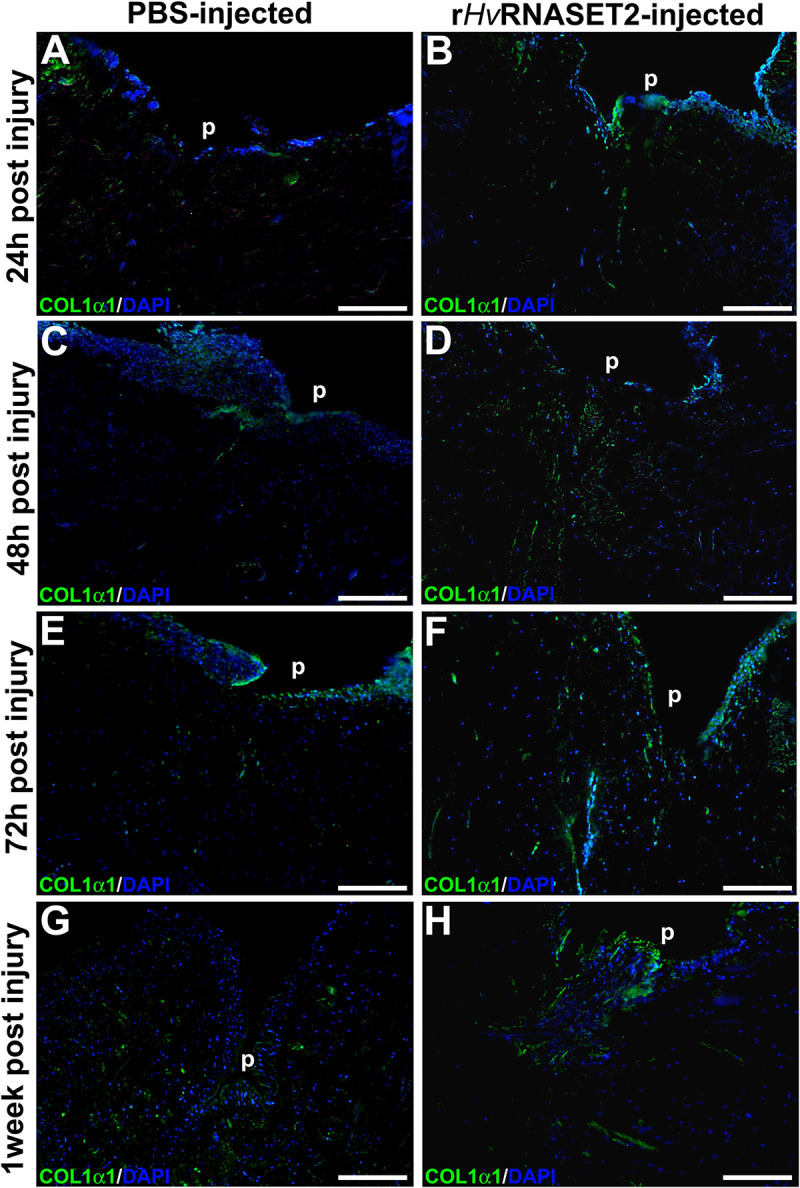
Immunofluorescent analyses of injured and PBS or r*Hv*RNASET2-injected leeches. The primary anti-COL1α1 antibody highlights a different synthesis of collagen between the two different treatments. Injured and PBS-injected animals show a lower detectable signal **(A,C,E,G)**, while in injured and r*Hv*RNASET2-injected leeches, an abundant production of collagen is clearly detectable **(B,D,F,H)**. Cell nuclei are stained in blue with DAPI. p: pseudoblastema, ECM: extracellular matrix. Bars in panels **(A–H)** 100 μm.

**FIGURE 6:**
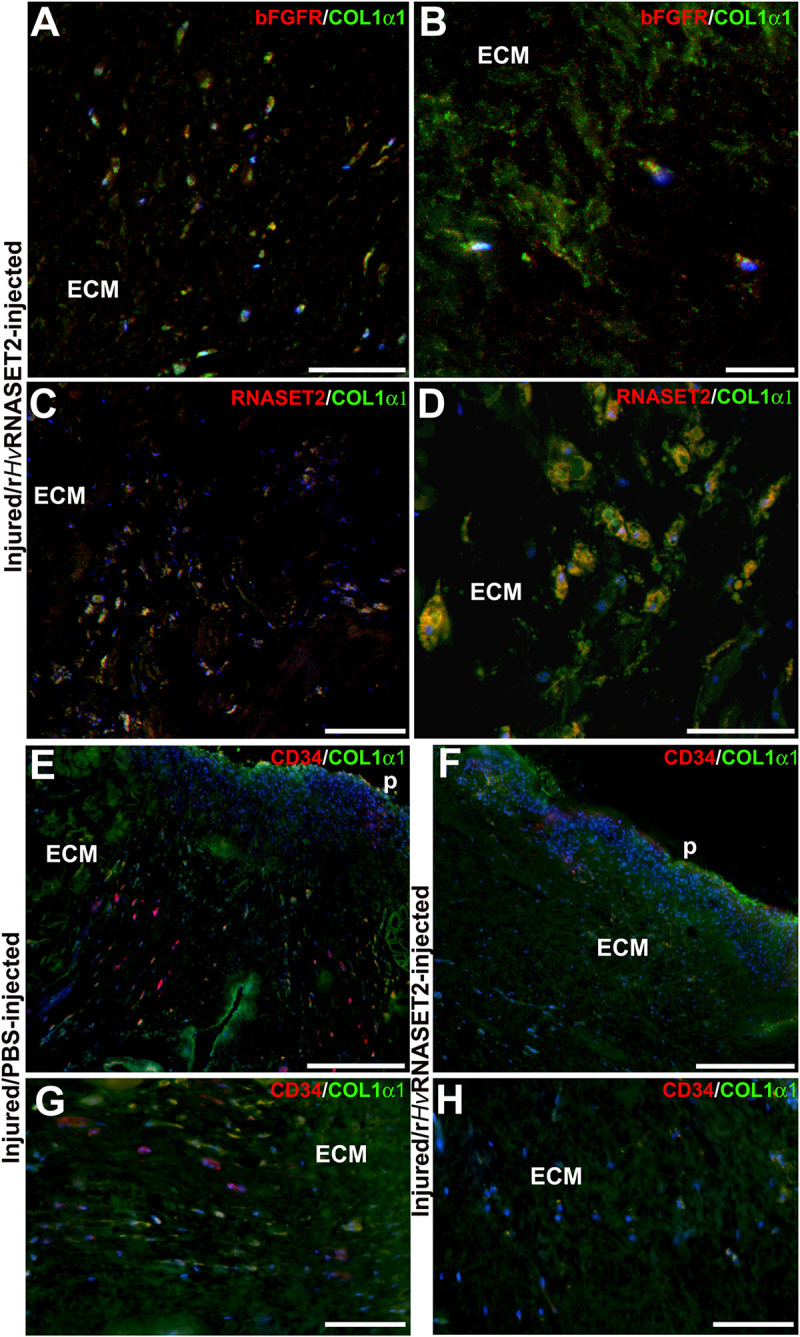
Double immunolocalization of collagen1-α1 (in green) and bFGFR/RNASET2 (in red) of wounded and r*Hv*RNASET2-injected leeches **(A–D)**, and COL1α1 (in green) and CD34 (in red) of wounded and PBS- or r*Hv*RNASET2-injected animals **(E–H)** 48 h from injury. Numerous activated bFGFR^+^ fibroblasts are visible in the lesioned tissue, which are involved in new collagen I fibrils production **(A,B)**. Several cells appear positive for both RNASET2 and collagen1-α1 antibodies **(C,D)**, suggesting a direct correlation between *Hv*RNASET2 and collagen I. Moreover, numerous CD34^+^ HSPCs are found in PBS-injected leeches **(E,G)** compared to r*Hv*RNASET2-treated animals **(F,H)**. Nuclei are stained in blue with DAPI. p: pseudoblastema, ECM: extracellular matrix. Bars in panels **(A,C–E)** 100 μm; bars in panels **(B,F,G)** 50 μm; bar in panel **(D)** 20 μm.

To better characterize the composition of newly synthesized ECM during the wound healing phases and to evaluate the role of *Hv*RNASET2 in inducing collagen neo-synthesis, immunofluorescence assays were performed (Figure 5). In the body wall of wounded and PBS-injected leeches (Figures 5A,C,E,G), a lower detectable marker collagen I signal was displayed at different time points from injuries, whereas the production of collagen I progressively increased in r*Hv*RNASET2-treated animals (Figures 5B,D,F,H), as also demonstrated by the presence of numerous bFGFR-expressing fibroblasts in the same area (Figures 6A,B). Moreover, the immunolocalization of *Hv*RNASET2 definitively correlated the increased levels of collagen I with a higher expression of this enzyme. As expected, *Hv*RNASET2 signal was higher in the r*Hv*RNASET2-injected area after 48 h, due not only to recombinant protein injection, but also to r*Hv*RNASET2-induced recruitment of more fibroblasts (Baranzini et al., 2020b), which in turn produced new collagen I (Figures 6C,D). Furthermore, only a few CD34 positive cells were visible in the injured and r*Hv*RNASET2-treated leeches (Figures 6E,H) after 48 h, compared to those seen in control animals (Figures 6E,G). This finding suggested that the reduced recruitment of myeloid precursors was correlated with the absence of new blood vessel formation. No signals were detected in the negative control experiments in which the primary antibodies were omitted (Supplementary Figures 1A–E).

### qPCR and Western Blot Analyses

**FIGURE 7:**
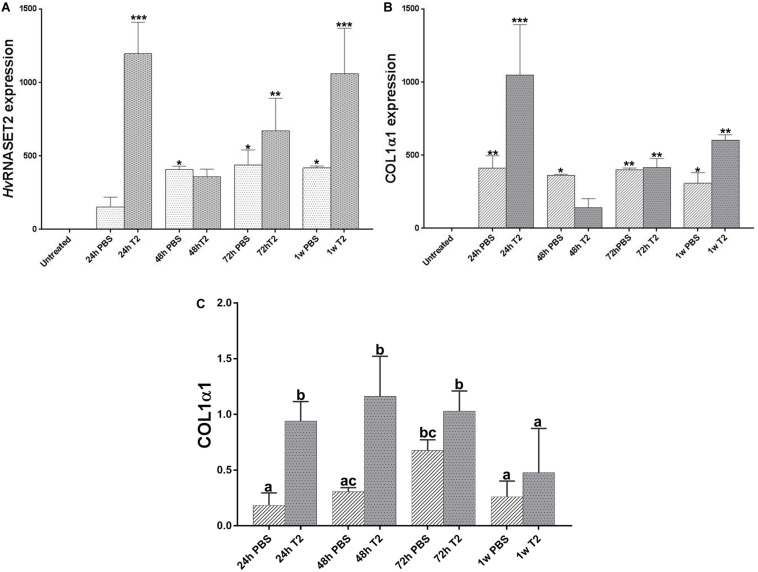
Graphs showing qRT-PCR analyses related to *Hv*RNASET2 **(A)** and COL1α1 **(B)** mRNA expression in PBS or r*Hv*RNASET2-injected leeches. The graphs represent *Hv*RNASET2 and COL1α1 quantification relative to the expression of the endogenous gene GAPDH. The mRNA expression fold change of treated animals is compared with that of untreated leeches, used as control. Statistical differences were calculated by one-way ANOVA and *p* < 0.05 was considered statistically significant. Western blot analyses on wounded and PBS- or r*Hv*RNASET2-injected leeches **(C)**. The graph illustrates the expression levels of collagen1α1 at different time points, calculated by densitometric analysis. The values are reported as relative optical density of the bands normalized to glyceraldehyde 3-phosphate dehydrogenase (GAPDH). Means with different letters indicate significant difference between treatments at different times. *P < 0.5, **P < 0.1, ***P < 0.01.

The *Hv*RNASET2 expression levels and the rate of neosynthesized collagen I were also assessed by means of real-time qPCR and western blot assays at different timings (Figure 7). As shown in the qPCR graphs, in both PBS- and T2-injected leeches the expression levels of both *Hv*RNASET2 (Figure 7A) and collagen I (Figure 7B) was higher than the untreated leeches, used as control. However, the *Hv*RNASET2 gene appeared to be expressed at already significantly higher levels after 24 h, subsequently decreasing at 48 h, and then reaching a new peak after 1 week. By contrast, *Hv*RNASET2 expression levels in PBS-treated animals remained rather constant. A similar result in *Hv*RNASET2-treated leeches was observed for collagen I, whereby a high expression peak was observed after 24 h, subsequently decreased after 48 h, but then raised again, reaching maximum values at 1 week from treatment. It is worth noting that collagen I expression values were also constant in all timings in wounded and PBS-injected leeches. This evidence was also validated by western blot analyses (Supplementary Figure 1F) showing the presence of 140 kDa bands (corresponding to the molecular weight of collagen I). As visible in the graph (Figure 7C), a greater deposition of collagen I appeared more visible in r*Hv*RNASET2-injected animals, compared to PBS-injected samples.

## Discussion

After physical damages, inflammation represents the preliminary step necessary to restore both the precise original structure and the correct homeostasis of tissues. Injuries induce a sudden physiological change in the lesioned microenvironment, in which the release of different cytokines and growth factors promotes the activation of many enzymatic cascades and consequent cellular reactions (Mescher et al., 2017). The importance of a rapid immune response is essential to accurately coordinate the subsequent events and to prevent any incorrect outcomes, both in vertebrates and in invertebrates (Tettamanti et al., 2003b; de Eguileor et al., 2005). Moreover, besides the local immune reaction, the activated cells are involved in the production of new ECM components, which constitute the fundamental scaffold for the migration of inflammatory precursors, the formation of blood vessels, and the restoration of the original architecture (Grimaldi et al., 2006). Given the clinical importance in investigating the existing correlation between inflammation and wound healing events, the use of alternative animals models, such as the medicinal leech, piqued interest, improving not only the knowledge of many preserved biological processes, but also of the molecules that take part in them, which appear to be evolutionarily conserved (Mescher et al., 2017).

In this context, the leech *H. verbana*, showing several similarities with vertebrates, represents a suitable invertebrate model that allows us to add novel insights to tissue regeneration. Moreover, in leeches, it has been recently demonstrated that the *Hv*RNASET2 enzyme not only regulates the inflammatory response, recruiting macrophages toward challenge tissues, but also plays a pivotal role in connective tissue remodeling, triggering fibroblasts activation and ECM reorganization (Baranzini et al., 2020b). This evidence and the pleiotropic nature of T2 ribonucleic enzymes indicate that this protein acts as a key molecule that can coordinate both inflammation and regenerative events. To confirm this hypothesis, injured and PBS- or r*Hv*RNASET2-injected leeches were evaluated by means of morphological and molecular assays.

Compared to undamaged leeches (Baranzini et al., 2017, 2019), light and TEM analyses revealed that in PBS-injected control animals, after wounding several cells migrated to the lesioned area during the earlier healing phase, confirming that the inflammatory response also characterizes the regenerative process in leeches. Moreover, the reorganization of the connective tissue, due to an accentuate fibroplasia, allows for angiogenetic events and the appearance of new blood vessels. These mechanisms are regulated by both macrophages and fibroblasts, which increased around the pseudoblastema and were fundamental for the development of correct wound repair. Like in vertebrates (Wynn and Barron, 2010; Mescher et al., 2017), macrophages are considered to be the main effectors of inflammatory response and fibrosis participating in the elimination of apoptotic cells and cellular debris in leeches as well. These cells, characterized by filopodia and blebs, which are probably involved in driving the migration and cell-to-cell signaling of macrophages in the connective tissue (Ma and Baumgartner, 2013; Eom, 2020), are derived from hematopoietic myeloid precursors, which originate in the botryoidal tissue and migrate to the lesioned area toward the neo-vessels. The beginning of vasculogenesis, which involves the early formation of the vascular system, is characterized by a marked remodeling of the botryoidal tissue that in turn represents a mechanism for the fast recruitment of a large number of cells involved in immune defense, wound healing, and regeneration. These myeloid precursors possess the ability to activate diverse genetic programs in response to peculiar environmental stimulation and give rise to immune cells and/or to vessel-associated myoendothelial cells involved in muscle regeneration (Grimaldi et al., 2009; Grimaldi, 2016). In parallel, fibroblasts appear elongated and star-shaped, characterized by the presence of multiple cytoplasmic laminae projections stretching into the extracellular space, near which new collagen fibril deposition becomes evident. As in vertebrates, where fibroblasts act with other mesenchymal cells to release different molecular effectors (such as monocyte chemoattractant and prostaglandin E2) in order to recruit macrophages and regulate monocyte activation (Bernardo and Fibbe, 2013; Mescher et al., 2017), this also occurs in leeches where these bFGF^+^ cells might promote both the inflammatory state and its subsequent resolution, directly producing new collagen I. Thus, in the medicinal leech, the continuous interaction between macrophages, fibroblasts, and other immune or mesenchymal cells avoids fibrotic events and promotes muscle regeneration and lesion repair after 1 week from injury, allowing the correct formation of the pseudoblastema and the complete closure of the wound.

Conversely, the overexpression of r*Hv*RNASET2 in the wounded area induces not only a significant increase in macrophages and type I granulocytes recruitment, confirming the ability of this enzyme to recruit various immunocompetent cells into the injured tissue (Baranzini et al., 2017, 2019), but also induces a massive deposition of ECM components, mainly evident 72 h post-injury. The deposition of new collagen I is due to the activation of numerous *Hv*RNASET2^+^/bFGF^+^ fibroblasts, as also demonstrated by molecular analyses. Especially in wounded and RNASET2-injected leeches, both *Hv*RNASET2 and collagen I gene expression levels appeared to be already significantly increased 24 h from treatment, which then decreased and reached a new peak after 1 week.

Strikingly, unlike control PBS-injected leeches, in injured *Hv*RNASET2^+^-injected animals a few vessels and muscle fibers were found in the newly formed connective tissue. These results lead us to hypothesize that although *Hv*RNASET2 promotes the formation of a solid collagen scaffold, its anti-angiogenic effect, already demonstrated in vertebrates (Acquati et al., 2011), might affect the number of CD34^+^ myoendothelial precursor cells recruited in the regenerating area. Thus, the incorrect regeneration of muscle tissue might be attributable to the failure to recruit these cells, which in physiological conditions are instead transported by the neo-vessels into the wound healing area and give rise to new muscle fibers (Grimaldi et al., 2009, 2010). Our hypothesis seems to be supported by the low number of vessels and by the considerably reduced angiogenesis process. Furthermore, in the wounded *Hv*RNASET2-injected leeches, the botryoidal tissue shows a typical inactivated form, arranged in cords or clusters.

## Conclusion

Understanding the events combining innate immune response and regeneration have gained considerable importance in recent years as it is now clear that these two processes are closely related and are fundamental for tissue homeostasis. Indeed, it is well known that a reduced tissue regeneration capacity is linked to a reduced inflammation process. It is therefore becoming essential to understand how the immune system is able to regulate the correct restoration of tissues, in order also to develop new strategies in the therapeutic field.

Our study carried out in a medicinal leech alternative animal model, in addition to avoiding the use of vertebrates for research, could lay the foundation for the development of new clinical strategies by highlighting *Hv*RNASET2 as a possible regulator of ECM remodeling during wound healing. This enzyme, by inducing the activation of fibroblasts, promotes the synthesis of new collagen I and ECM remodeling. As a secondary effect, a reduction of the regeneration of muscle tissue occurs, due to the probable anti-angiogenic effect of this enzyme. We speculate that lower vascularization prevents the migration of muscle precursors normally vehiculated by the vessels in the regenerating area.

## Data Availability Statement

The original contributions presented in the study are included in the article/Supplementary Material, further inquiries can be directed to the corresponding author/s.

## Author Contributions

AG and FA conceived the experiments, and wrote and edited the manuscript. NB and LP equally contributed to this work performed the experiments, and statistical analyses. NB prepared the original draft of the manuscript and all the figures. GT edited the manuscript and provided expertise. All authors critically reviewed the manuscript.

## Conflict of Interest

The authors declare that the research was conducted in the absence of any commercial or financial relationships that could be construed as a potential conflict of interest.

## Footnotes

1 http://rsbweb.nih.gov/ij/download.html

## Supplementary Material

Supplementary Figure 1 Immunolocalization (negative control) **(A–E)**. No signals are visible in anti-RNASET2 **(A)**, anti-COL1a1 **(B)**, CD34/COL1a1 **(C)**, bFGFR/COL1a1 **(D)**, and RNASET2/COL1a1 **(E)** negative control immunolocalizations, in which primary antibodies are omitted. Western blot immunoreactive bands of undamaged, injured, and PBS- or r*Hv*RNASET2-injected leeches, analyzed after 24, 48, 72 h, and 1 week post-treatment **(F)**. The anti-collagen I and anti-GAPDH antibodies detected in a leech tissue-specific immunoreactive band of about 140 and 37 kDa respectively, according to the molecular weight ladder (kDa).
