# Supplementary figures and images for: HvRNASET2 Regulate Connective Tissue and Collagen I Remodeling During Wound Healing Process
Source: Front Physiol. 2021 Feb 24;12:632506. doi: 10.3389/fphys.2021.632506 (PMC7943632; doi:10.3389/fphys.2021.632506)

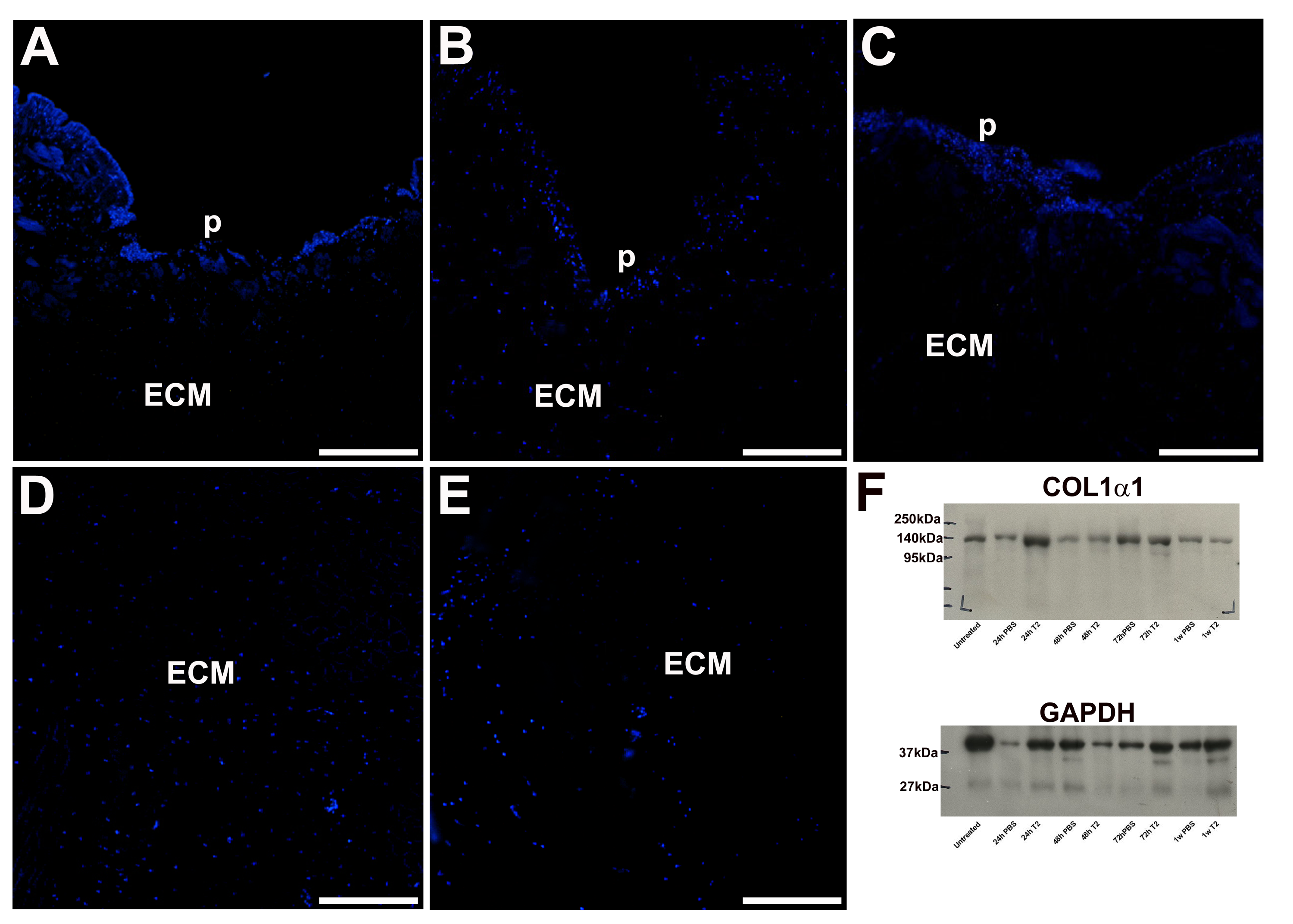

Supplement: Supplementary Figure 1 — Immunolocalization (negative control) (A–E). No signals are visible in anti-RNASET2 (A), anti-COL1a1 (B), CD34/COL1a1 (C), bFGFR/COL1a1 (D), and RNASET2/COL1a1 (E) negative control immunolocalizations, in which primary antibodies are omitted. Western blot immunoreactive bands of undamaged, injured, and PBS- or rHvRNASET2-injected leeches, analyzed after 24, 48, 72 h, and 1 week post-treatment (F). The anti-collagen I and anti-GAPDH antibodies detected in a leech tissue-specific immunoreactive band of about 140 and 37 kDa respectively, according to the molecular weight ladder (kDa). [file Image_1.TIF]
